# White Matter Hyperintensity Load Modulates Brain Morphometry and Brain Connectivity in Healthy Adults: A Neuroplastic Mechanism?

**DOI:** 10.1155/2017/4050536

**Published:** 2017-08-03

**Authors:** Matteo De Marco, Riccardo Manca, Micaela Mitolo, Annalena Venneri

**Affiliations:** ^1^Department of Neuroscience, University of Sheffield, Sheffield, UK; ^2^Functional MR, S.Orsola-Malpighi Hospital, Department of Biomedical and Neuromotor Science (DIBINEM), Bologna, Italy

## Abstract

White matter hyperintensities (WMHs) are acquired lesions that accumulate and disrupt neuron-to-neuron connectivity. We tested the associations between WMH load and (1) regional grey matter volumes and (2) functional connectivity of resting-state networks, in a sample of 51 healthy adults. Specifically, we focused on the positive associations (more damage, more volume/connectivity) to investigate a potential route of adaptive plasticity. WMHs were quantified with an automated procedure. Voxel-based morphometry was carried out to model grey matter. An independent component analysis was run to extract the anterior and posterior default-mode network, the salience network, the left and right frontoparietal networks, and the visual network. Each model was corrected for age, global levels of atrophy, and indices of brain and cognitive reserve. Positive associations were found with morphometry and functional connectivity of the anterior default-mode network and salience network. Within the anterior default-mode network, an association was found in the left mediotemporal-limbic complex. Within the salience network, an association was found in the right parietal cortex. The findings support the suggestion that, even in the absence of overt disease, the brain actuates a compensatory (neuroplastic) response to the accumulation of WMH, leading to increases in regional grey matter and modified functional connectivity.

## 1. Introduction

The adult human brain retains the capability for structural and functional modifications. This ability, labelled “neuroplasticity,” is the outcome of multiple mechanisms acting at numerous levels, including the cellular and synaptic levels [1], which may result in changes detectable using neuroimaging techniques and data modelling [2]. The study of neuroplasticity has been associated with different types of research. Some studies have explored *induced* neuroplastic changes, through the exploitation of “manoeuvrable” mechanisms in clinical populations (e.g., physical therapy, noninvasive brain stimulation, deep-brain stimulation, neuropharmacology, aerobic exercise, and cognitive rehabilitation). Other studies have investigated the *automatic* processes of neuroplasticity triggered by damage of neural tissue, as is seen after not only acute events (like stroke or traumatic brain injury [3]) but also accompanying chronic neurodegenerative conditions (such as Alzheimer's disease [4]). The mechanisms of plasticity, however, are not unique to clinical populations but are relevant for people who simply undergo the physiological processes of ageing and even young adults. A large number of studies have tested experimental hypotheses based, for instance, on cognitive interventions or motor training in healthy adults, to test for induced neuroplasticity (e.g., [5]). At the same time, some effort has also been made to characterise automatic compensatory instances associated with neural and cognitive ageing. This has been extensively studied in association with task-fMRI paradigms focused on differences in activation between younger and older adults. Although a proportion of older adults maintains levels of behavioural performance indistinguishable from those of young adults, it has been noted that this is achieved in association with a tendency to recruit more prefrontal regions during memory and perceptual tasks [6]. Along similar lines, high-performing older adults also tend to show more bilateral patterns of activation, and this has been seen in the prefrontal cortex [7], as well as in the parietal lobes [8]. These compensatory increases in activation to support performance on a task can be considered as due to processes of neuroplasticity [9]. Similar changes have also been seen in paradigms of resting-state functional connectivity, with age-dependent changes (decreases or increases) observed in the posterior and anterior default-mode network [10] and with increased levels of network-to-network interaction [11]. In all likelihood, these changes are compensatory to maintain function in the face of adverse events associated with ageing, such as functional dedifferentiation [12], cerebral atrophy [13], and subcortical white matter damage. Usually of vascular or inflammatory nature [14], the white matter hyperintensities (WMHs) frequently seen in the brain of otherwise healthy adults cause decreases in interneuronal speed of processing and connectivity [15]. As WMHs accumulate, compensatory mechanisms maintain function. When compensation is no longer effective, and reserve is depleted, cognitive function declines. When WMH accumulation generates symptoms, it may put a strain on various cognitive functions [16–18], although mechanisms of reserve may mitigate this trend [19]. A number of studies have shown compensatory task overactivation in the presence of WMHs [20, 21], but no research has yet addressed the association between WMHs and “positive” changes in resting-state functional connectivity, secondary to compensatory plasticity. In addition, there are studies showing positive associations between WMH load and cortical thickness in patients with small-vessel disease and Alzheimer's disease [22, 23], indicating that the neural system reacts to the presence of WMH by inducing a neuroplastic response which may be adaptive or detrimental. Although the presence of WMHs predicts decrements of global grey matter volumes in healthy adults [24], no study has yet focused on regional morphometry.

In this study, we analysed brain images from a sample of neurologically healthy adults who had undergone structural and resting-state fMRI and a comprehensive neuropsychological assessment. We quantified the amount of acquired WMH load using an automated procedure, and we tested the positive association between WMH load and (1) grey matter volume and (2) resting-state network connectivity, with voxel-by-voxel methods.

## 2. Materials and Methods

### 2.1. Participants

Fifty-one datasets acquired on healthy volunteers (35 females) were analysed retrospectively to address the research question. Under standard hypothesis-testing conditions (i.e., *α* = 0.05; power = 0.8), this sample size is sufficiently large to detect a significant bivariate correlation associated with a moderate effect size [25].

The demographic characteristics of the participants are illustrated in Table 1. All participants were enrolled in this study as healthy volunteers, at the Royal Hallamshire Hospital, Sheffield (United Kingdom). Neurological screening was carried out at recruitment on each participant aged 48 or above, to rule out the presence of major neurological symptoms. In addition, each participant completed a comprehensive battery of neuropsychological tests to ascertain the absence of cognitive deficits. Table 1 also reports the descriptive statistics associated with the performance obtained on tests which assess cognitive domains susceptible to ageing and neurodegeneration, namely, abstract-semantic reasoning (the similarities subtest of the WAIS battery), verbal and visuo-spatial memory (the Prose Memory test and the delayed recall of the Rey-Osterrieth complex figure, resp.), visuo-constructional skills (the copy of the Rey-Osterrieth complex figure), inhibition and shifting abilities (the Stroop and the Trail-Making test, resp.), and lexical production (the Letter Fluency test). Since the error interference on the Stroop test was limited (mean = 0.24, standard deviation = 0.90, and 14 errors made in total within the entire sample), the time interference index was the only effect calculated for this test [26].

Ethical approval for the procedures described in this study was obtained from the Yorkshire and Humber Regional Ethics Committee, Reference number: 12/YH/0474. Informed signed consent was obtained from all volunteers. All experimental procedures were strictly in compliance with the Declaration of Helsinki (1964).

### 2.2. MRI Image Acquisition

An experimental MRI session was completed by each participant. The protocol included anatomical and resting-state functional acquisitions obtained with a Philips Ingenia 3T scanner. Three MRI sequences were used in this study: a three-dimensional T1-weighted image (voxel size: 0.94 mm × 0.94 mm × 1.00 mm; TR: 8.2 s; TE: 3.8 s; FOV: 256 mm; matrix size: 256 × 256 × 170), a three-dimensional T2-weighted fluid-attenuated inversion recovery (FLAIR) magnetic resonance imaging sequence (voxel size: 0.56 mm × 0.56 mm × 0.56 mm; TR: 4.8 s; TE: 0.20 s; matrix size: 448 × 448 × 326), and a resting-state echo planar BOLD scan (voxel size: 1.80 mm × 1.80 mm × 4.00 mm; TR: 2.6 s; TE: 35 s; FOV: 230 mm; number of slices: 35; minimum scan duration: 5 minutes and 25 seconds). Participants were instructed to lay supine and remain as still as possible for the entire duration of the MRI protocol. Dummy scans were acquired prior to the resting-state sequence to obtain electromagnetic equilibrium.

### 2.3. T1-Weighted Image Processing

The processing and modelling pipeline was run using Matlab R2014a (Mathworks Inc., UK) and Statistical Parametric Mapping (SPM) 12 software (Wellcome Trust Centre for Neuroimaging, London, UK). The T1-weighted images were segmented to compute individual native space maps of grey matter, white matter, and cerebrospinal fluid [27]. Volumetric quantifications of each tissue class were then carried out using the “get_totals” script (http://www0.cs.ucl.ac.uk/staff/g.ridgway/vbm/get_totals.m). The total intracranial volume was calculated by summing up the volumetric quantification of all three tissue classes. Grey matter and white matter fractions were then computed as a ratio between each tissue volume and the total intracranial volume. These anatomical indices are reported in Table 1 for descriptive purposes. In addition, voxel-based morphometry procedures were run [27] for a voxel-by-voxel analysis of the association between WMH load and regional grey matter volumes. Briefly, maps of grey matter were normalised to the SPM 12 T1-weighted template and smoothed with an 8 mm full width at half maximum Gaussian kernel.

### 2.4. T2-Weighted Image Processing

The Lesion Segmentation Tool was used to segment the WMHs. Originally, this toolbox was developed to segment white matter lesions that are normally found in patients with multiple sclerosis [28]. Subsequent research, however, has reliably utilised it in samples of healthy elderly adults, or adults with cardiovascular risk factors [29–31]. First, this instrument processes the T1-weighted image to create a partial volume estimation image, then coregisters T1-weighted and FLAIR images, and carries out an inverse warping of the white matter tissue map. Subsequently, the FLAIR image intensity distribution is computed for the three tissue classes, and a native space lesion map is created. A threshold of 0.3 was used for this purpose [28]. Importantly, the quantification of WMHs does not differentiate between periventricular and deep WMHs. At the end, the total amount of WMHs (expressed in ml) was divided by the total intracranial volume and was then multiplied by 100 to obtain a ratio expressing WMH load as a percentage of intracranial volume [29].

### 2.5. Resting-State fMRI Image Processing

Resting-state functional images were preprocessed via a standardised pipeline. This included the following: (1) initial slice timing, conceived to assign homogenous temporal properties to slices within each volume; (2) spatial realignment, devised to “stack up” all volumes within each run and, thus, correct for volume-to-volume spatial displacement; (3) spatial normalisation, designed to register each native space acquisition to the default SPM 12 echo planar template in the Montreal Neurological Institute space; (4) temporal band-pass filtering (0.01 Hz to 0.1 Hz), applied to minimise the impact of nonneurogenic sources of variability on the BOLD signal rhythmicity [32] and achieved using the REST toolbox [33]; and finally (5) spatial smoothing (6 mm) was set up to maximise the signal-to-noise ratio. A quality check was carried out on each image prior to and during the preprocessing steps to rule out the presence of unexpected signal artefacts, excessive in-scanner motion, or normalisation errors.

An independent component analysis was then run on the complete group of 51 smoothed outputs. This type of inferential model assumes that the variability seen in the pattern of observable variables is the result of a linear combination of independent signalling sources [34]. An independent component analysis extrapolates a number of latent and statistically independent variables, which account for the pattern of observable variability with an optimal goodness of fit. The GIFT toolbox was used for this purpose [35], and the Infomax principle was chosen, setting the number of independent components to be estimated at 20.

**Figure 1:**
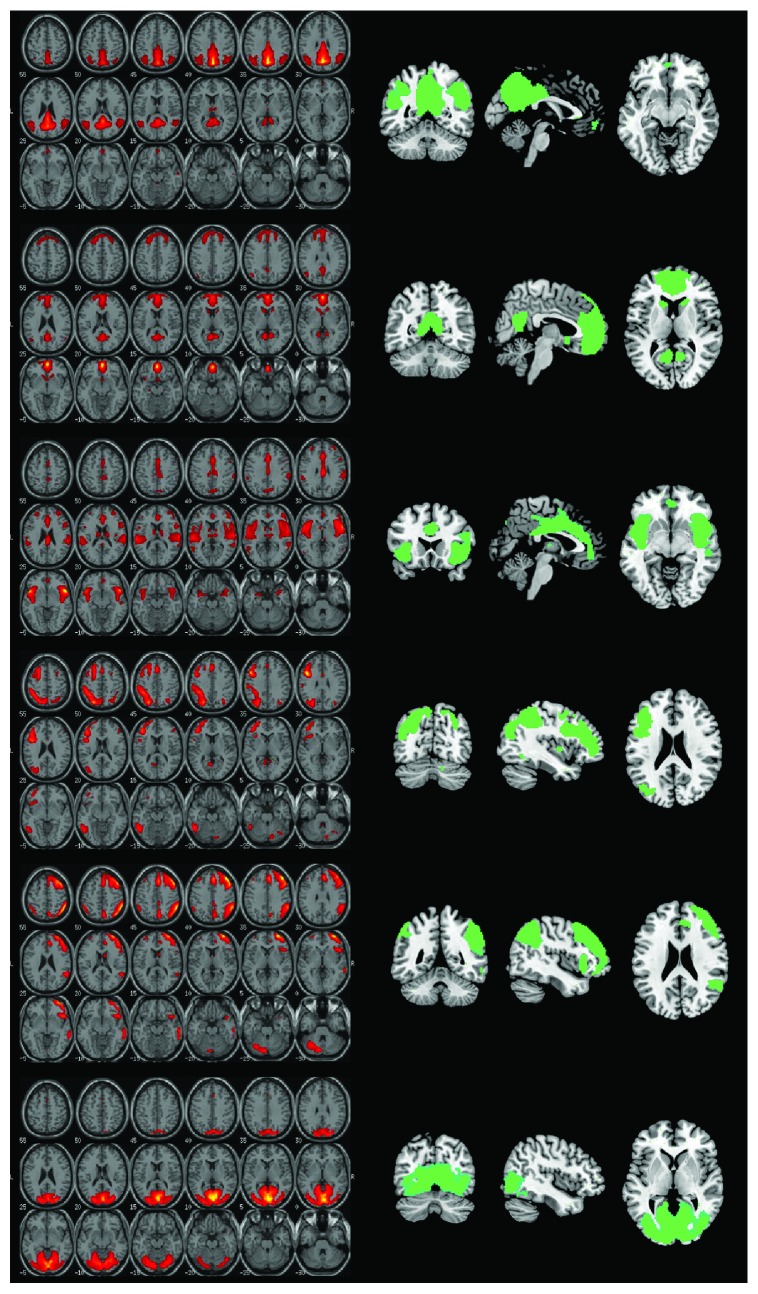
The six haemodynamic networks selected for this study, top to bottom: posterior default-mode network, anterior default-mode network, salience network, left frontoparietal network, right frontoparietal network, and visual network. The output of the independent component analysis is illustrated on the left, with maps expressing the *z* scores of each component. These same maps are shown on the right side as the output of a one-sample *t*-test carried out on the entire group.

The maps of six prominent networks were chosen (Figure 1). These were the anterior and posterior default-mode network, the salience network, the left and right frontoparietal networks, and, as a methodological control, the visual network. Subject-specific networks expressed as three-dimensional maps of *z* scores represented the dependent variable of the inferential models.

### 2.6. Statistical Modelling

Nonparametric correlations were run to characterise the association between white matter damage and each of the continuous variables included in Table 1. Linear models were run to test the association between WMH load and grey matter/network connectivity. The multiple-regression option on SPM was used for this purpose. Since the scope of the study was to describe the mechanisms of adaptive plasticity, we focused on the positive association contrasts (i.e., more grey matter, or more connectivity, in association with more subcortical load). Given the strong association found between WMH load on one hand and age and global levels of atrophy estimated by grey matter fraction on the other hand (see Results section), these two variables were included in all models as nuisance regressors. These covariates were also added in order to model the exclusive effects of WMHs independently of the level of atrophy and, importantly, to follow a methodology that would allow us to rule out the possibility that findings may simply be due to general effects of the ageing trajectory. Furthermore, since brain and cognitive reserve can influence the pattern of neural structure and functional connectivity, total grey matter volume and education level were included as additional covariates. For all models, the significance threshold was set at *p* < 0.005 (uncorrected) at the set level and *p* < 0.05 (family-wise error corrected) at the cluster level. Result coordinates were converted into the Talairach space using a nonlinear transformation (http://imaging.mrc-cbu.cam.ac.uk/downloads/MNI2tal/mni2tal-m) and localised using the Talairach Daemon client [36].

## 3. Results

**Figure 2:**
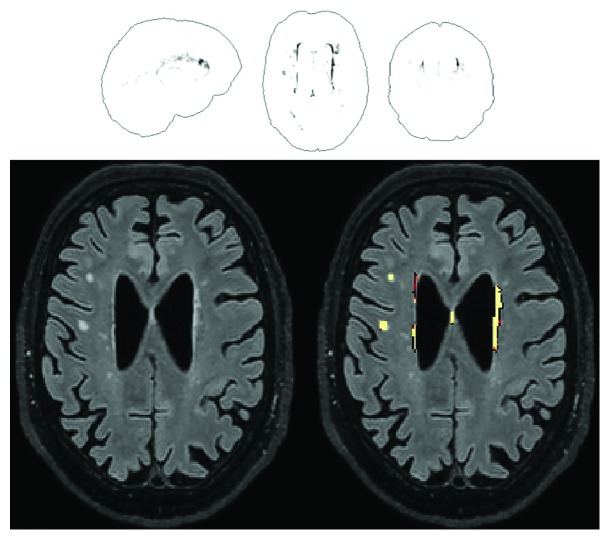
An example of the output from the Lesion Segmentation Tool for the quantification of WMHs. An overview is given in the glass-brain template shown in the upper half. A single slice is instead reproduced in the lower half, showing mainly periventricular, but also some sparse deep WMHs. This specific participant is a 70-year-old man with a raw WMH volume of 3.25 ml, equal to 0.21% of his intracranial volume.

The raw amount of WMH lesions ranged from 0 to over 32 ml, equivalent to 0% to 2.17% of individual total intracranial volume (see Figure 2 for an illustrative example). Of the entire set of variables included in Table 1, WMH load correlated significantly with age (*Spearman's rho* = 0.848, *p* < 0.001), total grey matter volume (*Spearman's rho* = −0.692, *p* < 0.001), grey matter ratio (*Spearman's rho* = −0.732, *p* < 0.001), immediate recall on the Prose Memory test (*Spearman's rho* = −0.360, *p* = 0.009), delayed recall on the Prose Memory test (*Spearman's rho* = −0.306, *p* = 0.029), and the Stroop time interference index (*Spearman's rho* = 0.532, *p* < 0.001). Since it is well established that age and education levels do influence performance on episodic memory and inhibitory skills, additional partial correlation models were run between WMH load and cognitive performance on these three measures, controlling for age and education. The association with memory scores was no longer significant, but the association with the Stroop time interference index survived. This association was still significant even after further correcting for global levels of atrophy.

**Figure 3:**
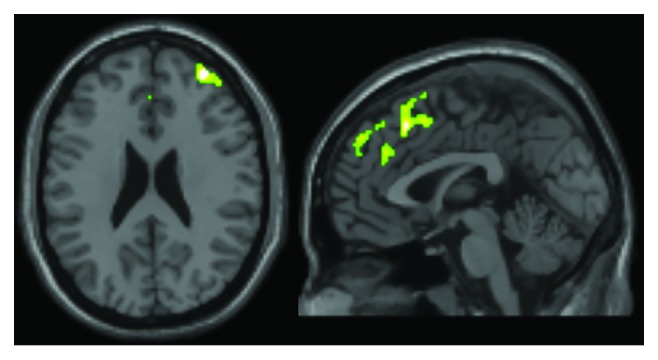
The positive association between WMH load and grey matter volumes.

Positive associations were found between WMH load and the voxel-based model of grey matter. These were found in the anterior part of the brain, specifically in the right anterior prefrontal cortex and in a large portion of the medial prefrontal cortex and cingulate gyrus (Table 2; Figure 3).

**Figure 4:**
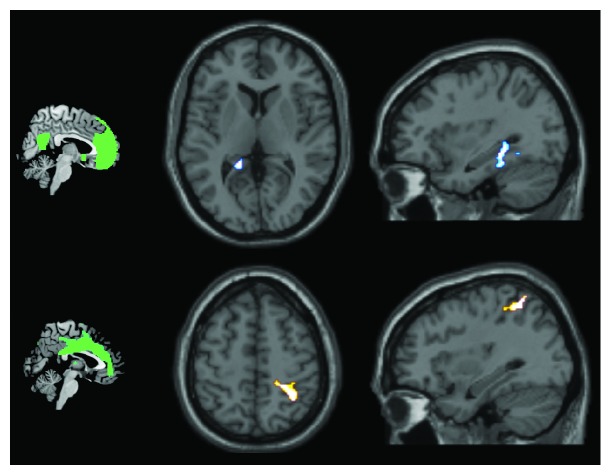
The positive association between WMH load and network connectivity (anterior default-mode network in the upper half and salience network in the lower half). The two networks are illustrated on the left.

The only two networks in which positive associations were found were the anterior default-mode network and the salience network (Table 2; Figure 4). Within the anterior default-mode network, increased connectivity correlated with increased WMH load in the left mediotemporal complex, including the posterior cingulate, the parahippocampal gyrus, and the posterior part of the hippocampus. Within the map of the salience network component, a significant association was found in the right parietal lobe, in the primary sensory cortex, and in the superior and inferior parietal lobules.

The average *z* scores for these two clusters were extracted for post hoc correlation models, in an attempt to characterise the results in more detail. In an explorative way, correlation models were run between network connectivity of these two clusters and each neurostructural and neuropsychological variable included in Table 1. The sole significant associations were those with white matter volume (anterior default-mode network: *p* = 0.010), white matter ratio (anterior default-mode network: *p* < 0.001; salience network: *p* = 0.039), and with the Stroop time interference index (anterior default-mode network: *p* = 0.080; salience network: *p* = 0.028). These last two *p* values dropped to 0.008 and 0.010, respectively, after controlling for age and level of education.

Finally, albeit beyond the scope of the study, post hoc models were run to test the presence of negative associations between WMH load and anterior default-mode network and salience network connectivity. No significant results were found.

## 4. Discussion

WMHs are relatively frequent among healthy adults, and, together with atrophy, they induce a decrease in the healthy neural tissue available for the computations necessary to sustain cognitive functioning. Nonetheless, the brain pursues adaptive plasticity to compensate for the age-associated accumulation of white matter damage. This can take the form of computational “scaffolds” as theorised by the Scaffolding Theory of Ageing and Cognition, indicating that additional neural circuitry is recruited in compensation for brain damage to support cortical functions [37]. On these grounds, in this study, we specifically explored the positive associations between WMHs and functional connectivity of the typical main resting-state networks to clarify which pathways of connectivity are upregulated when lesions accumulate. We constructed an inferential model which could test the “clean” association between WMHs and those neural variables of interest, controlling for both global levels of atrophy (i.e., grey matter fraction) and age. Although a strong negative association was found between WMH load and, both, grey matter volume and grey matter fraction, indicating potential collinearity issues between these aspects, a recent study reported that the statistical association between WMHs and cognition is independent of levels of atrophy [38]. This finding justifies the use of global levels of atrophy as a correction factor, to control for their potential confounding effects. In a similar way, although WMH volume was also profoundly correlated with age, by no means can we limit the entire ageing trajectory to the sole accumulation of WMHs. As a consequence, the possibility that this would result in collinearity issues was ruled out on theoretical grounds.

Out of the six networks analysed, only the anterior default-mode network and salience network showed significant results. The significant cluster within the anterior default-mode network was located in a hub that is normally a key part of the default-mode network [39]. Since increased connectivity of the anterior default-mode network with the posterior cingulate has already been reported in association with ageing [10], it is possible that this increase indicates a preferential avenue of compensation achieved by the system to fight against multiple sources of neural damage. The default-mode network (expressed as a single or double component) has to deactivate when the person engages in a task. It is possible that the changes in connectivity seen in response to ageing, or to specific types of neural insult such as those resulting in WMHs, are necessary to safeguard appropriate and efficient task deactivation. Since our findings simply indicate the presence of a statistical association, any interpretation based on a cause-effect mechanism remains merely speculative.

For the salience network, the cluster was located between regions supporting primary sensory elaboration and regions of high-order perceptual processing, which in part overlap with the parietal portion of the right frontoparietal network. The salience network is usually active in the transitional state between task deactivation and task activation [40] and is involved in stimulus selection for behaviour guidance. In general terms, the process of ageing causes a general downregulation of the salience network, with reduced internetwork connectivity between the salience network and the frontoparietal network [41], a finding which goes in the opposite direction of that found in this study. It could be argued, however, that the brain can cope with a limited amount of damage (i.e., that due to mild WMH load) by upregulation of the salience network-frontoparietal network connectivity, but it is not equally successful in limiting all of the detrimental changes induced by the complex process of ageing (e.g., including progressive accumulation of WMH and atrophy).

A positive association was also found in the anatomy of the forebrain. The largest cluster included a portion of the medial prefrontal cortex and the anterior cingulate cortex (Figure 3). This territory is part of the salience network (the posterior part of the cluster) and the anterior default-mode network (the anterior part of the cluster). From an interpretational viewpoint, this is consistent with the findings of the functional connectivity analysis models, as the same cerebral territory appears to be affected by WMH load, structurally and functionally.

Analysis of the effects of WMH load on cognitive performance yielded results consistent with our findings discussed above. The only score that showed a significant association with WMH load was the Stroop time interference index. Evidence has shown that this task is profoundly dependent on the default-mode network and the salience network. As the default-mode network deactivates while the person engages in a Stroop task, hubs of the salience network activate [42]. In our study, this association retained its significance even after correcting for age, education level, and global level of atrophy. This strongly indicates that the executive skills of inhibition and interference resolution are the cognitive domains most susceptible to the presence of WMHs. Interestingly, the regional connectivity expressed at the level of the two clusters emerging from the anterior default-mode network and salience network models was positively correlated with the Stroop time interference index (i.e., more connectivity is needed when interference is overcome in a longer time). On these grounds, we interpret these significant correlations as indicative of an adaptive neuroplastic increase of connectivity in response to the accumulation of WMHs.

Although an anatomical-functional convergence of findings can be extrapolated across modalities, it remains to be clarified why the connectivity of certain networks was not influenced by WMH load. Interestingly, in a previous study carried out on healthy controls and patients suffering from vascular or Alzheimer dementia, WMHs were found to affect the function of the frontal lobe irrespective of their location in the template [43]. Although the authors speculated that this might be due to a prefrontal convergence of white matter tracts, the ultimate reason why this occurs remains unknown. It is interesting to note, however, that we confirmed the preferential influence of WMH load on frontal lobe function and structure.

Despite the neat pattern of findings, which defines a common direction for volumetric, connectivity, and cognitive variables, it is more challenging to delve into the actual mechanisms by which neuroplastic modifications might occur. It was suggested that, aside from reducing the amount of neural tissue, atrophy also acts as an active inductor of plasticity, because age-dependent overactivations are typically seen in those regions which show prominent age-dependent shrinkage [44]. It is possible that the gradual accumulation of WMHs would have a similar active function fostering the stimulation of neuroplastic grey matter adaptations and increases in functional connectivity. Longitudinal studies are needed to address this issue, potentially following up populations of patients who are particularly at risk of developing WMHs (e.g., individuals with one or more cardiovascular risk factors) over time.

Another interpretational point deserves attention. If an authentic causative association existed between WMH load and an increase in prefrontal and antero-limbic grey matter, it would be necessary to clarify its biological correlates. In such case, two major candidate mechanisms might be at play: synaptogenesis and vascular changes [2]. An increase in the number of synapses would be consistent with the evidence of increased functional connectivity, while an increase in regional vasculature would be consistent with the aetiology of the damage, as the vascular system would compensate for the ischaemic blockages that generate WMHs.

This study, however, is not free from limitations. Although previous findings showed that WMHs seem to have a preferential impact on frontal function regardless of their location [43], it would be informative to characterise each single lesion with additional qualitative and topographical details (i.e., whether it was a periventricular or deep WMH and which tracts were affected). This is an important aspect to be considered as part of future studies. A second limitation is that it is not possible to derive a cause-effect link from the pattern of statistical associations. To address this, a prospective longitudinal study might provide the missing interpretational link (i.e., studying the extent to which differences in WMH load from baseline to retest predict any change in brain structure and functional connectivity from baseline to retest).

## 5. Conclusion

In summary, significant positive associations were found between the brain WMH load and the volume and functional connectivity of the anterior default-mode network and the salience network. The WMH load was also associated with executive function, specifically inhibition skills, measured with the Stroop time interference index. Positive associations were found between these abilities and the connectivity within the significant anterior default-mode network and salience network clusters. This convergence suggests that WMHs may be linked to neuroplastic changes in the frontal and antero-limbic territories in healthy adults. The tendency of prefrontal and antero-limbic regions to be positively responsive to subcortical damage is an aspect that deserves attention. In fact, similar (but, in all likelihood, less effective) mechanisms may be involved in the structural and functional reorganisation following more serious and extensive brain damage. On this note, rehabilitation programmes might benefit from evidence showing that certain regions of the brain are particularly prone to compensatory changes.

## Figures and Tables

**Table 1:** Demographic, morphometric, and cognitive characterisation of the sample enrolled in this study.

Variable	Mean	SD	Median	Minimum	Maximum
*Demographic features*					
Age (years)	61.98	16.42	63.00	26.00	100.00
Education (years)	14.88	3.19	15.00	10.00	22.00
*Subcortical damage*					
WMH load (ml)	3.26	5.83	0.82	0.00	32.58
*Global neurostructural indices*					
Grey matter volume (ml)	636.28	67.20	633.01	446.74	752.21
White matter volume (ml)	415.07	44.29	408.88	337.43	527.50
Total intracranial volume (ml)	1457.69	157.06	1439.66	1217.24	1855.43
Grey matter fraction	0.44	0.06	0.45	0.33	0.56
White matter fraction	0.29	0.02	0.29	0.22	0.34
*Cognitive scores*					
WAIS—similarities	24.96	4.17	26.00	15.00	32.00
Prose Memory test—immediate recall	15.98	3.52	16.00	9.00	24.00
Prose Memory test—delayed recall	19.20	2.76	19.00	14.00	25.00
Rey-Osterrieth complex figure—copy	32.23	2.96	33.00	23.00	36.00
Rey-Osterrieth complex figure—recall	15.95	4.83	16.00	7.00	27.00
Letter Fluency test	47.18	13.25	47.00	16.00	75.00
Stroop test—time interference (s)	20.89	14.22	16.50	3.00	84.00
Trail-Making test—part B minus part A (s)	37.75	33.74	30.00	0.00	223.00

One participant did not complete the Stroop test because of colour blindness. One participant, aged 100 years, did not complete the Rey-Osterrieth complex figure, the Stroop test, or the Trail-Making test because of significant macular pathology. SD: standard deviation.

**Table 2:** Positive association between WMHs and brain structure and functional connectivity.

Cluster-level *p*FWE	Cluster extent (voxels)	*z* score at local maximum	Brodmann area	Side	Brain region	Talairach coordinates		
						*x*	*y*	*z*
*Regional grey matter volume*								
0.045	875	4.67	10	R	Superior frontal gyrus	33	51	22
		4.59	10	R	Middle frontal gyrus	45	47	16
		3.69	10	R	Inferior frontal gyrus	46	52	1
		3.64	10	R	Superior frontal gyrus	34	57	16
		3.33	10	R	Superior frontal gyrus	26	56	3
		3.22	10	R	Superior frontal gyrus	30	61	6
<0.001	3149	4.67	8	R	Superior frontal gyrus	10	43	44
		4.54	32	L	Medial frontal gyrus	−6	12	45
		4.50	8	R	Superior frontal gyrus	12	37	46
		3.98	6	R	Superior frontal gyrus	10	23	61
		3.96	8	L	Medial frontal gyrus	−2	25	43
		3.82	9	L	Medial frontal gyrus	−4	50	34
		3.74	6	L	Superior frontal gyrus	−18	22	56
		3.69	32	R	Middle cingulate cortex	3	36	28
		3.59	6	R	Superior frontal gyrus	2	26	56
		3.35	8	L	Superior frontal gyrus	−14	36	53
		3.33	6	L	Superior frontal gyrus	−20	17	62
		3.21	9	L	Superior frontal gyrus	−6	56	27
*Anterior default-mode network*								
0.046	240	3.77	29	L	Posterior cingulate cortex	−16	−42	8
		3.51		L	Hippocampus	−26	−39	2
		3.40	37	L	Fusiform gyrus	−34	−36	−13
		3.38	36	L	Parahippocampal gyrus	−34	−32	−10
		3.29	19	L	Parahippocampal gyrus	−36	−49	−1
		3.23		L	Hippocampus	−32	−35	−7
		3.11		L	Hippocampus	−24	−37	−3
*Salience network*								
0.028	267	4.00	3	R	Postcentral gyrus	26	−34	55
		3.56	40	R	Inferior parietal lobule	34	−48	56
		3.31	7	R	Superior parietal lobule	26	−51	63
		3.26	40	R	Inferior parietal lobule	32	−40	52
		3.06	7	R	Superior parietal lobule	32	−51	62
		3.02	3	R	Postcentral gyrus	20	−30	59

L: left; R: right; FWE: family-wise error.
